# Evaluating survey methods for bat roost detection in ecological impact assessment

**DOI:** 10.1111/acv.12574

**Published:** 2020-04-02

**Authors:** J. S. P. Froidevaux, K. L. Boughey, C. L. Hawkins, G. Jones, J. Collins

**Affiliations:** ^1^ School of Biological Sciences University of Bristol Bristol UK; ^2^ Université de Toulouse, INRAE, UMR DYNAFOR Castanet‐Tolosan France; ^3^ Bat Conservation Trust London UK

**Keywords:** Chiroptera, day‐roost, detection probability, environmental impact assessment, sampling effort, synanthropic species, survey methods, bat roost

## Abstract

The disturbance, damage and destruction of roosts are key drivers of bat population declines worldwide. In countries where bats are protected by law, bat roost surveys are often required to inform ecological impact assessments. Yet, evidence‐based information on survey methodology to detect bat roosts is crucially lacking, and failing to detect a roost can lead to serious errors during decision‐making processes. Here, we assess the efficacy of bat roost surveys in buildings as implemented in the UK. These consist of a daytime inspection of buildings, followed by a series of acoustic surveys at dusk/dawn if during the daytime inspection evidence of bats is found, or if the absence of bats cannot be verified. We reviewed 155 ecological consultants’ reports to (1) compare survey outcome between daytime inspection and acoustic surveys and (2) determine the minimum sampling effort required during acoustic surveys to be confident that no bats are roosting within a building. We focused on two genera of bats most frequently found in buildings in Europe – *Pipistrellus* (crevice roosting species with high‐intensity echolocation calls that can be easily detected by ultrasound detectors) and *Plecotus* (species that roost in open spaces and which emit faint echolocation calls that are difficult to detect). Daytime inspections were efficient in detecting open‐roosting species such as *Plecotus* species but were likely to miss the presence of crevice‐dwelling ones (here *Pipistrellus* species) which may lead to erroneous conclusions if no acoustic surveys are subsequently prescribed to confirm their absence. A minimum of three and four acoustic surveys are required to be 95% confident that a building does not host a roost of *Pipistrellus* species and *Plecotus* species, respectively, thus exceeding current recommendations. Overall, we demonstrated that reports submitted as part of an ecological impact assessment provide suitable data to test and improve survey methods.

## Graphical abstract

Missing the presence of a bat roost during an ecological impact assessment can lead to serious errors during the decision‐making process and may result in harm or disturbance to bats or destruction of their roosts, and therefore to a criminal offence in countries where bats are legally protected. Here, we reviewed a sample of ecological consultants’ reports submitted for planning purposes in the United Kingdom, to determine optimal survey methodology and sampling effort associated with bat roost surveys in buildings. We found that the implementation of different but complementary methods—i.e. daytime inspection of a building followed up by acoustic surveys at dusk/dawn— is paramount to detect bat roosts in buildings. We also estimated the minimum sampling effort required to detect bat presence in buildings when using acoustic methods and provide recommendations."

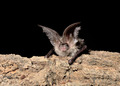

## Introduction

In temperate ecosystems, most insectivorous bats use sheltered structures as day‐roosts (Kunz, 1982; Dietz, von Helversen & Nill, 2007). Many roosts are in human‐made structures such as buildings, hence many bat species are referred to as synanthropic, living alongside humans (Russo & Ancillotto, 2015; Voigt *et al.*, 2016). The loss or scarcity of natural roosts, as well as the suitable conditions and environment provided by buildings for roosting, have contributed to the increased use of the latter (Brigham, 1991). Buildings provide protection from predators, shelter from adverse weather conditions and favourable microclimatic conditions for reproduction and rearing young (Lausen & Barclay, 2006). Furthermore, buildings offer a diversity of potential roosts, from spacious areas such as attics that can be suitable for bats that roost in open spaces, to a variety of small interstices suitable for crevice‐dwelling species. However, bats roosting in buildings are at greater risk of conflict with human demands (Stone, Jones & Harris, 2013; Russo & Ancillotto, 2015; Stone *et al.*, 2015). The disturbance and destruction of roosts have been identified as significant drivers of bat population declines that are believed to have occurred during the 20th century (Stebbings, 1988), and still represent threats to the survival of local populations (Hutson, Mickleburgh & Racey, 2001). Preventing the disturbance, damage and destruction of roosts located in buildings is therefore crucial to the conservation of synanthropic bat species (Fenton, 1997; Voigt *et al.*, 2016).

Bats are legally protected across Europe under the European Union Habitats Directive (92/43/EEC), in an attempt to prevent further population declines. There is slight variation in the legislation across countries but essentially it is an offence to disturb or harm a bat or to damage, destroy or obstruct access to a place used by a bat for shelter and protection (i.e. a roost). In the UK, in situations where an offence against bats may be committed, for example during development, it is possible to apply for a special derogation licence (otherwise known as a mitigation licence or European Protected Species licence) to allow activities to proceed legally. The licence application requires information from an ecological impact assessment that include findings of a series of bat roost surveys, and a proposed strategy to avoid, mitigate or compensate for impacts on bats such as disturbance or damage to/destruction of roosts. Since the ecological impact assessment is used to inform decision‐making processes (Mandelik, Dayan & Feitelson, 2005), it is crucial to ensure that roost surveys are of high accuracy. This is particularly true as there is growing evidence that ecological impact assessments may fail to reduce bat mortality caused by anthropogenic disturbances (e.g. wind turbines; Lintott *et al* *.*, 2016). There is a need for robust science in ecological impact assessment studies to ensure data accuracy and reliability (Green *et al.*, 2016).

Bat roost surveys of buildings are routinely carried out by ecological consultants to inform development control (planning) and European Protected Species licensing. These surveys aim to discover whether bats are present or absent and, if present, the species, numbers, roost type and access points (Collins, 2016). Bat roost surveys in the UK consist of a preliminary roost assessment (PRA), which is a daytime inspection of the interior and exterior of a building to detect live or dead bats and evidence of bat occupation such as droppings or feeding remains. If evidence of bats is found or there are locations in the building where bats and evidence of bats could be hidden, the PRA is usually followed up with a series of emergence and re‐entry surveys (ERSs). These consist of surveyors standing outside the building at dusk and dawn, respectively, to watch and listen for bats departing from or returning to their roosts, with the aid of ultrasonic bat detectors. Emergence and re‐entry surveys can only be carried out during the seasons when bats are active, and, if there is a possibility that maternity colonies are present, best practice is to carry out at least some of the surveys during the spring/summer months of May–August (Collins, 2016).

Missing the presence of a bat roost or failing to identify the roost type (e.g. maternity colony) during a bat roost survey can lead to serious errors during the decision‐making process and may result in harm, disturbance or destruction of bats and their roosts, and therefore to a criminal offence under domestic legislation arising from the Habitats Directive. Bat species differ in their detectability within roosts, both visually, due to differences in their roosting ecology (e.g. crevice‐dwelling vs. open‐roosting species) and acoustically, due to differences in echolocation call design (e.g. low‐ vs. high‐intensity echolocation calls). Thus, variation in detection probabilities arising from (1) the survey method implemented and (2) the sampling effort deployed should be carefully considered when designing roost surveys, while optimizing the time and money spent. Despite the extensive scientific literature on the improvement and optimization of bat surveys (e.g. Flaquer, Torre & Arrizabalaga, 2007; Skalak, Sherwin & Brigham, 2012; Stahlschmidt & Bruhl, 2012; Froidevaux *et al.*, 2014; Law *et al.*, 2015; Froidevaux, Fialas & Jones, 2018; Richardson *et al.*, 2019), specific recommendations regarding roost surveys are scarce (Fleming *et al.*, 2013; Chambers *et al.*, 2015) and mostly restricted to the estimation of colony size (Kloepper *et al.*, 2016).

The aim of this study is to assess and suggest improvements to survey methodology to detect bat roosts in buildings during the ecological impact assessment process. Our objectives were to first compare the outcome of the PRA to the subsequent ERSs and, second, determine the minimum sampling effort required during the ERSs to be reasonably confident (at 95%) that no bats are roosting within a building. Our predictions are as follows: (1) open‐roosting species would be more likely to be detected during a PRA than crevice‐dwelling ones given that the PRA is done by visual inspection; (2) species that are difficult to detect acoustically would be less detectable during ERSs since these surveys are conducted using ultrasonic bat detectors; (3) maternity roosts of crevice‐dwelling species would be missed if only PRAs were carried out; (4) the implementation of both survey methods – PRA and ERSs – would lead to the discovery of a higher number of bat species than the PRA on its own; (5) a higher number of visits would be required to establish the absence of species difficult to detect acoustically compared with species emitting intense and easily identifiable echolocation calls. We used *Pipistrellus* spp. (*Pipistrellus pipistrellus* and *P. pygmaeus*) and *Plecotus* sp. (*Plecotus auritus* or *P. austriacus*, but almost certainly always *P. auritus* given the rarity of *P. austriacus* in the UK) as examples to test predictions 1, 2, 3 and 5 given that these two taxonomic groups are the most frequently encountered during roost surveys in the UK. *Pipistrellus* spp. are known to roost in small crevices located either inside or outside of buildings (Dietz *et al.*, 2007), emerge just after sunset (Rydell, Entwistle & Racey, 1996) and have relatively intense and distinguishable echolocation calls (Holderied & von Helversen, 2003). In contrast, *Plecotus* sp. roost in open areas in buildings (Entwistle, Racey & Speakman, 1997; Dietz *et al.*, 2007) have late emergence and early re‐entry times (Rydell *et al.*, 1996), and emit faint echolocation calls (Waters & Jones, 1995). Thus, our choice of species allows direct testing of our predictions since *Pipistrellus* spp. are relatively difficult to detect within roosts visually, but relatively easy to detect visually and acoustically using bat detectors as they are emerging and returning; whereas *Plecotus* sp. are relatively easy to detect within roosts visually, but difficult to detect visually and acoustically as they are emerging and returning. Since the methodology used in the UK to detect bat roosts in buildings is one of the most advanced worldwide, our findings are of relevance globally and may help in designing surveys to detect bats roosting in other types of structures.

## Materials and methods

### Data origin, sampling design and variable extraction

We accessed bat survey reports from ecological impact assessments that were conducted for planning applications between 2007 and 2015 in two English counties, Hampshire and Warwickshire. These counties contain a mix of rural, suburban and urban areas which permitted us to cover a wide range of representative cases. We divided the counties by Local Planning Authorities (public authorities that undertake planning functions for a particular area) because each authority has a different approach to the consideration of biodiversity in planning and we wanted to ensure there was a representative sample of cases from each. Only Local Planning Authorities having more than 10 reports from both the PRA and subsequent ERSs were retained (five in Hampshire and four in Warwickshire).

We implemented a hierarchical stratified random sampling design to select the bat survey reports. We randomly selected between 14 and 19 reports within each pre‐selected Local Planning Authority of each county. For each report, we extracted 23 variables (Table 1). From the PRA, we extracted the date of the survey, the presence or absence of bats and, where relevant, the species of bats found, numbers of individuals, function of each roost and the type of evidence used for species identification. For the ERSs, we extracted the following for each individual survey: survey date, survey type, weather conditions (temperature, cloud cover, wind speed and rain), survey effort (numbers of surveyors and duration of survey), presence or absence of bats, species of bats found, number of new species found, number of individuals, and function of each roost discovered (see full description in Table 1). To avoid compiler bias, all the data were extracted by the same person (lead author) and entered into a Microsoft Access database.

**Table 1:** List of variables extracted from the ecological impact assessment reports

	Variable	Description
General information	Location	County, Local Planning Authority, and UK grid reference of the building surveyed
	Building type	Type of building surveyed: house, group of houses, barn, industrial building, hospital, school or other
	Ecological consultancy	Name of the ecological consultancy that undertook the survey
Preliminary roost assessment (PRA)	Date	Date of the survey
	Presence of bats	Yes or no
	Species name	Scientific name of the species or genus present. We noted as Chiroptera spp. when the identification was not possible
	Number of species	Number of species present
	Number of individuals	Number of individuals present per species
	Roost function	Day‐roost, maternity roost or night‐roost per species present
	Type of evidence	Type of evidence regarding the presence and the identification of the bats detected: live animal, dead animal, droppings, DNA analysis of droppings. Note that in this study areas with only remains of insects such as moth wings were not considered to be bat roosts but rather feeding perches
Emergence and re‐entry surveys (ERSs)	Date	Date of each survey
	Survey type	Dusk emergence or dawn re‐entry survey
	Temperature	Temperature at the start of the survey (°C)
	Cloud cover	Cloud cover at the start of the survey: none (<5%), partially cloudy (5–49%), cloudy (50–79%), overcast (80–100%)
	Wind speed	Wind speed at the start of the survey (Beaufort scale)
	Rain	Presence of rain during the survey: none, light rain or heavy rain
	Number of surveyors	Number of people that undertook the survey
	Sampling duration	Number of sampling hours
	Presence of bat	Yes or no
	Name of the species	Scientific name of the species or genus present. We noted as *Chiroptera* spp. when identification was not possible
	Number of new species	Number of new species present compared to (1) PRA and (2) other visits that took place during ERSs
	Number of individuals	Number of individuals present per species
	Roost function	Day‐roost, maternity roost or night‐roost per species present

### Statistical analysis

All the analyses were conducted using R 3.3.3 (R Development Core Team, 2017) using the ‘lme4’ (Bates *et al.*, 2015), ‘unmarked’ (Fiske & Chandler, 2011), ‘AICcmodavg’ (Mazerolle, 2017), and ‘MuMIn’ (Bartoń, 2016) packages.

#### Objective 1: PRA versus ERSs

We fitted a series of generalized linear mixed models to (1) investigate whether the discovery of individual bats and maternity colonies within a building differs between the PRA and the subsequent ERSs and (2) test if conducting ERSs after a PRA provides additional information on number of species present in a building. We considered the presence of *Pipistrellus* spp. and *Plecotus* sp. individuals (predictions 1 and 2), presence of maternity colonies (prediction 3) and bat species richness (cumulative number of all bat species detected in the building; prediction 4) as response variables while survey method (PRA vs. ERSs) was included in the models as an explanatory variable. To test prediction 3, we pooled together maternity colonies of all species encountered, as too few maternity colonies were discovered during the surveys in our sample to permit taxon‐level analysis. Since the number of surveys differs between the two survey methods (generally one survey conducted during PRA vs. several surveys during ERSs), the outcomes of each individual survey undertaken during ERSs were pooled together. To take into account the hierarchical stratified random sampling design, building IDs nested within Local Planning Authorities, nested with counties were introduced as random factors. We used a binomial distribution when looking at individual bat and maternity presence/absence and a Poisson distribution for models on species richness. The explanatory variable was considered as statistically significant if the 95% confidence intervals of its estimate did not overlap zero (Nakagawa & Cuthill, 2007).

#### Objective 2: estimation of bat detection probabilities and minimum survey effort required during acoustic surveys

We used site‐occupancy models developed by MacKenzie *et al. *(2002) to estimate detection probabilities (*p*) of bats during the ERSs. We built single‐species single‐season models considering the assumptions of the models mostly fulfilled: (1) occupancy state at each building is static over surveys within the sampling season; (2) detection of bats and detection histories at each building are independent; (3) bats are identified correctly and (4) there is no heterogeneity in detection probability. The sampling protocol applied in the original reports enables us to fully satisfy the three last assumptions since surveyors use ultrasonic acoustic devices to detect and identify bat species and we assume that consultants applied best‐practice approaches following UK bat survey guidelines (Bat Conservation Trust, 2007; Hundt, 2012; Collins, 2016). The first assumption is, however, more difficult to meet as single individuals as well as maternity colonies of the species we studied often switch roost during the season (Feyerabend & Simon, 2000; Fleischmann & Kerth, 2014). Model outputs were therefore interpreted with caution.

Detection/non‐detection history of *Pipistrellus* spp. and *Plecotus* sp. was introduced as response variables into the models. Each building was treated as a site and each emergence or re‐entry survey as an independent visit. As the number of ERS surveys varied considerably amongst studies, we selected the first four surveys only. Since we were not interested in assessing factors that may influence bat occupancy in buildings but rather obtaining robust estimation of detection probabilities, only buildings with known bat occupancy (*ψ*) during ERSs were selected (*Pipistrellus* spp.: *N* = 79; *Plecotus* sp.: *N* = 43). This method has been successful in identifying sources of variation in detection probabilities of other animals (e.g. Murn & Holloway, 2016). We included five explanatory variables in our models that may influence bat detection probabilities: temperature, cloud cover, number of surveyors, survey type and sampling duration (see Table 1 for more details). Wind speed and amount of rain were disregarded from the analyses as most of the surveys were conducted during relatively calm (<3 in Beaufort scale) and dry nights. Continuous variables were standardized beforehand (i.e. rescaled to the same unit) and collinearity between all variables was checked using either Spearman’s rank correlation test or chi‐square test of independence depending on the nature of the variables (continuous or categorical); no correlation was found. In total, we produced for each response variable a set of 32 models, that is, all possible models including the most complex as well as the null ones.

We applied an information‐theoretic approach using the Akaike information criterion corrected for small sample size (AICc; Burnham & Anderson, 2002) to select the most parsimonious models. Goodness of fit of the most complex models was assessed using bootstrap analysis (MacKenzie & Bailey, 2004) with 999 replicates. We used quasi‐AICc values instead of AICc values to compare and rank models when lack of fit occurred (ĉ > 1). Model selection was performed using the *dredge* function. Finally, we undertook a model‐averaged procedure following the so‐called zero method (shrinkage towards zero) of the top 2QAICc of models (Burnham & Anderson, 2002) to account for model selection uncertainties (Grueber *et al.*, 2011) and to obtain model‐averaged estimates of detection probabilities and associated standard errors. The significance of the effect of each variable was assessed through 95% confidence intervals (Nakagawa & Cuthill, 2007).

To test prediction 5, we evaluated the minimum number of sampling surveys (*N*_min_) required to be 95% confident that a bat is absent from a building using the model‐averaged estimates of detection probabilities (*p*): *N*_min_ = log(*α*)/log(1 − *p*) with *α* = 0.05 to represent the 95% confidence level (Kéry, 2002; Pellet & Schmidt, 2005). Furthermore, to construct detectability curves, we calculated the probability of detecting a bat if present as a function of number of surveys: *p_i_* = 1 − (1 − *p*)*^i^* with *p_i_* being the detection probability of a bat after *i* surveys (Wintle *et al.*, 2005).

## Results

We extracted data from 155 reports (83 in Hampshire and 72 in Warwickshire) from nine Local Planning Authorities. In total, the surveys were conducted by 50 different ecological consultancies, with two‐thirds of the reports coming from 10 of these. The surveys were carried out mainly in relation to the renovation/conversion of buildings: 72% involved houses, 19% barns and the remaining 9% were other types of building (e.g. primary schools, industrial buildings). Overall, *Plecotus* sp. (*P. auritus* or *P. austriacus*) were the most frequently encountered species during the initial PRA (48% of PRAs) while *Pipistrellus* spp. (*P. pipistrellus* and/or *P. pygmaeus*) were the most frequently detected species during the subsequent ERSs: this taxon was detected in 51% of the buildings surveyed during ERSs. Other species were also found roosting in the buildings surveyed: *Eptesicus serotinus* (12 buildings), *Myotis mystacinus*/*brandtii* (8), *M. nattereri* (7), *Rhinolophus hipposideros* (2) and *Barbastella barbastellus* (1).

### Objective 1: PRA versus ERSs

**Figure 1:**
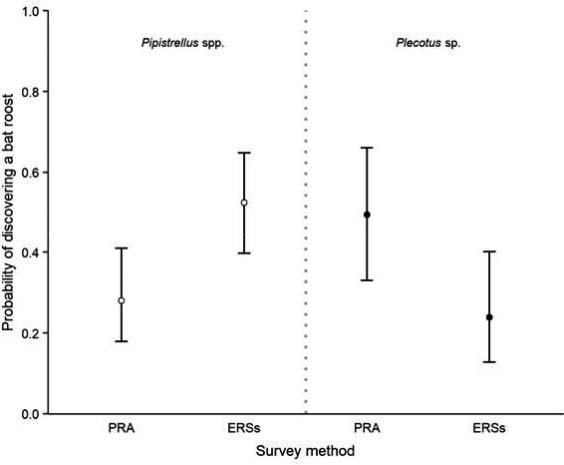
Estimated probabilities of discovering a bat roost within a building as function of survey method (PRA: preliminary roost assessment; ERSs: emergence and re‐entry surveys). Model predictions and associated 95% confidence intervals are represented by the circles and black solid lines, respectively. Open circles: *Pipistrellus* spp.; black filled circles: *Plecotus* sp.

**Figure 2:**
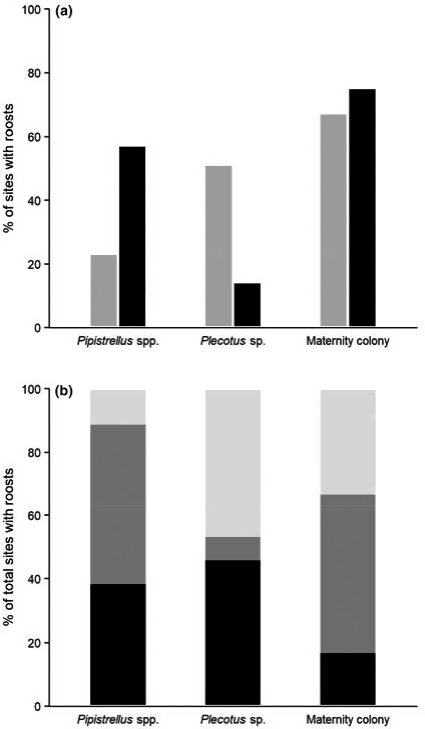
(a) (1) In grey the percentage of sites with roosts detected during the preliminary roost assessment (PRA), that were not then detected by the emergence and re‐entry surveys (ERSs); and (2) in black the percentage of sites with roosts detected during the ERSs, that had not previously been detected during the PRA. (b) The percentage of total sites with roosts that were (1) discovered during the PRA but not during the ERSs (light grey); (2) discovered during the ERSs but not during the PRA (dark grey) and (3) discovered during both PRA and ERSs (black).

The probability of discovering a bat roost within a building (predictions 1 and 2) was strongly affected by the survey method implemented (Table 2). Our models for *Pipistrellus* spp. and *Plecotus* sp. highlight a taxon‐specific pattern (Fig. 1). These models indicated that the ERSs are more likely to confirm the presence of *Pipistrellus* spp. within a building than the PRA while the opposite is true when looking at *Plecotus* sp. We found that ERSs failed to detect the presence of bat roosts (of any type) previously identified during the PRA in 23% of the cases for *Pipistrellus* spp. and in 51% of cases for *Plecotus* sp. Conversely, of the *Pipistrellus* spp. and *Plecotus* sp. roosts detected during the ERSs, 57% and 14% had not been detected during the PRA, respectively (Fig. 2).

**Table 2:** Estimates with associated standards errors (se) and lower and upper 95% confidence intervals (CI) of the generalized linear mixed models (GLMMs) relating to the effects of survey method (PRA, preliminary roost assessment; ERSs, emergence and re‐entry surveys) on bat presence and species richness

Response variable	Explanatory variable	Estimate (±se)	Lower 95% CI	Upper 95% CI
*Pipistrellus* spp. presence^a^	PRA versus ERSs	1.04 (±0.34)	0.37	1.71
*Plecotus* sp. presence^a^	PRA versus ERSs	−1.14 (±0.39)	−1.91	−0.37
Maternity colony presence^a^	PRA versus ERSs	0.99 (±1.04)	−1.05	3.03
Cumulative number of species^b^	PRA versus PRA & ERSs	0.48 (±0.10)	0.28	0.68

^a^ GLMMs with a binomial distribution.

^b^ GLMMs with a Poisson distribution.

We did not find any statistical evidence of an effect of survey method on the discovery of maternity colonies (prediction 3). Maternity colonies were encountered infrequently in our sample, being discovered at only 8% of sites. In total, 13 maternity colonies were discovered during surveys, including seven of *Plecotus* sp., five of *Pipistrellus* spp. and one of *E. serotinus*. Two colonies were located within the same site. Seven maternity colonies were discovered during the PRA, and a further six were discovered during ERSs after no evidence had been found during the PRA (Fig. 2). However, of those maternity colonies missed during the PRA two‐thirds (*N* = 4) were maternity colonies of *Pipistrellus* spp., as such 80% of all *Pipistrellus* spp. maternity colonies discovered in the sample were missed during the PRA.

Finally, the cumulative number of species recorded during both PRA and ERSs (prediction 4) was significantly higher than the species richness found during the PRA (Table 2). Thus, conducting ERSs provides a significant gain of information on number of species present within a building.

### Objective 2: detection probabilities and sampling effort

For both *Pipistrellus* spp. and *Plecotus* sp., temperature, sampling duration and number of surveyors were included in at least one of the most parsimonious models on detection probabilities (Table 3). However, none of these variables was significant given that confidence intervals of the estimates overlapped zero (Table 4). Similarly, we did not find any statistical evidence of the effects of cloud cover on detection probabilities of *Plecotus* sp. Survey type (dusk emergence vs. dawn re‐entry survey) did not affect bat detection probability; it was not retained in any of the most parsimonious models. Two‐thirds of the ERSs were conducted at dusk.

**Table 3:** Description of the full, null and most parsimonious site occupancy models (ΔQAICc < 2) built to estimate bat detection probabilities (*p*) of *Pipistrellus* spp. and *Plecotus* sp. during emergence and re‐entry surveys

Taxa	Model	*K*	QAICc	ΔQAICc	*ω_i_*
*Pipistrellus* spp.	*ψ*(.), *p*(Temperature, Sampling duration)	5	143.05	0.00	0.59
	*ψ*(.), *p*(Temperature, Sampling duration, No. of surveyors)	6	143.76	0.71	0.41
	*ψ*(.), *p*(Temperature, Sampling duration, No. of surveyors, Cloud cover, Survey type)	8	148.26	5.21	–
	*ψ*(.), *p*(.)	3	156.96	13.91	–
*Plecotus* sp.	*ψ*(.), *p*(Temperature)	4	94.98	0.00	0.38
	*ψ*(.), *p*(Temperature, No. of surveyors)	5	95.33	0.31	0.31
	*ψ*(.), *p*(Temperature, Cloud cover)	5	96.68	1.70	0.16
	*ψ*(.), *p*(Temperature, Sampling duration)	5	96.78	1.80	0.15
	*ψ*(.), *p*(Temperature, Sampling duration, No. of surveyors, Cloud cover, Survey type)	8	103.03	8.05	–
	*ψ*(.), *p*(.)	3	101.51	6.53	–

Model selection process was based on the quasi‐Akaike’s information criterion adjusted for small sample size (QAICc). The number of parameters (*K*), QAICc and delta QAICc are given for each model. AICc weight (*ω_i_*) is given for the most parsimonious ones only. The occupancy parameter (*ψ*) was fixed to 1. A full description of each covariate are found in Table 1.

**Table 4:** Standardized model‐averaged estimates with shrinkage, standard error (se) and 95% confidence intervals (CI) of the variables present in the most parsimonious models on bat detection probabilities (ΔQAICc < 2)

Response variable	Explanatory variable	Estimate (±se)	Lower 95% CI	Upper 95% CI
*Pipistrellus* spp.	Temperature	−0.12 (±0.22)	−0.54	0.31
	Sampling duration	−0.39 (±0.22)	−0.82	0.04
	No. of surveyors	0.13 (±0.22)	−0.31	0.56
*Plecotus* sp.	Temperature	0.12 (±0.27)	−0.40	0.65
	Sampling duration	0.01 (±0.11)	−0.20	0.22
	No. of surveyors	0.13 (±0.25)	−0.36	0.62
	Cloud cover	0.08 (±0.29)	−0.49	0.66

QAICc, quasi‐Akaike’s information criterion adjusted for small sample size.

**Figure 3:**
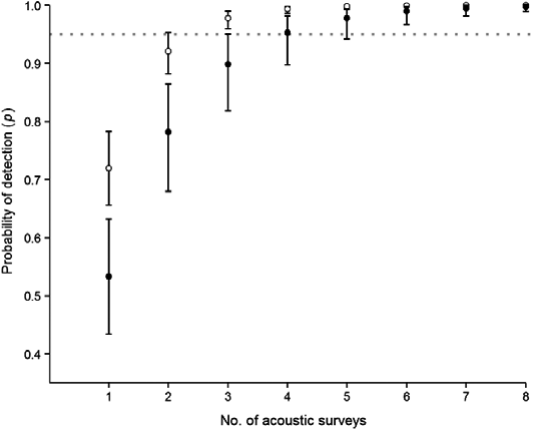
Estimated detection probabilities of *Pipistrellus* spp. (open circles) and *Plecotus* sp. (filled circles) as function of the number of emergence and re‐entry surveys. The black vertical bars represent the upper and lower 95% confidence intervals of the estimates while the grey horizontal dotted line indicates the 95% threshold (see Section Objective 2: estimation of bat detection probabilities and minimum survey effort required during acoustic surveys).

Model‐averaged estimates of detection probability varied among taxa (prediction 5; Fig. 3). When conducting a single‐visit ERS, the probability of detecting *Pipistrellus* spp. in the building was relatively high (*p* = 0.72). However, this probability is lower when targeting *Plecotus* sp. (*p* = 0.53). A minimum of three ERSs are required to be 95% confident that a building does not host a roost of *Pipistrellus* spp. To be certain at 95% that no roosts of *Plecotus* sp. are present, our results suggest that a minimum of four ERSs are necessary (Fig. 3).

## Discussion

We reviewed a sample of ecological consultant’s reports submitted for planning purposes in the UK to determine optimal survey methodology and sampling effort associated with bat roost surveys in buildings. We have shown that reports submitted as part of an ecological impact assessment provide suitable data with which to test survey recommendations. We found compelling evidence that the implementation of different but complementary methods – that is, daytime inspection of a building followed up by acoustic surveys at dusk/dawn – is paramount to adequately identify bat roosts in buildings. We also estimated the minimum sampling effort required to detect bat presence in buildings when using acoustic methods. Though we chose the UK as a case study, we believe that our findings and recommendations are of relevance globally since bats are legally protected across Europe and in many countries worldwide, and ecological impact assessment is a widespread process used to inform decision‐making. It is therefore important to provide guidelines not only to ecological consultants but also to stakeholders that are part of the decision‐making process to ensure that scientific standards are met (Mandelik *et al*., 2005). Even when no impacts are predicted, the results are still relevant in projects that aim to inventory bats roosting in human‐made structures.

### Daytime inspection versus acoustic surveys at dusk/dawn

We found marked differences in detection between open‐roosting bat species (*Plecotus* sp.) and crevice‐dwelling ones (*Pipistrellus* spp.) during the PRA: crevice dwellers were more likely to be missed from daytime inspection. While we highlighted that in almost half of the cases studied PRA failed to detect the presence of *Pipistrellus* spp. in a building, our findings may actually reflect an underestimation of what plausibly occurs. For our analysis, we deliberately retained only those reports presenting both PRA and subsequent emergence and re‐entry acoustic surveys, that is, reports in which ERSs were not prescribed by the assessment made during the PRAs were omitted from our analyses. Consequently, we do not know for these cases whether the building assessed during the PRA did actually host a bat roost, and we believe based on our results that is very likely that some roosts of *Pipistrellus* spp. were missed, even though the building presented very low potentiality according to surveyors. Due to their roosting ecology, crevice‐dwelling species are difficult to detect visually and may show no other obvious signs of occupation (e.g. droppings) when roosting in well‐hidden places (e.g. in cavity walls, between roof tiles, etc.). We used *P. pipistrellus* and *P. pygmaeus* as representatives of the crevice‐dwelling guild but our results can be extrapolated to other crevice dwellers that may use buildings as day‐roosts, namely *E. serotinus*, *M. brandtii*,* M. nattereri*, *M. mystacinus*, *Nyctalus leisleri* and *Pipistrellus nathusii* in the UK (Harris & Yalden, 2008).

Bat species vary in their detectability during ERSs, and differences in call intensity among bats partly explain these variations (Britzke, Gillam & Murray, 2013). Species that broadcast faint echolocation calls such as *Plecotus* sp. are less acoustically detectable than species producing high‐intensity echolocation calls (Russo, Ancillotto & Jones, 2017). Other call features may substantially influence bat detectability including call frequency. The likelihood of recording bats producing high‐frequency sounds (e.g. *Rhinolophus* spp.) will indeed be lower than that of recording species emitting low‐frequency calls (e.g. *E. serotinus*) as higher‐frequency calls undergo stronger attenuation (Lawrence & Simmons, 1982). Differences in emergence behaviour (early vs. late emergence) may also influence bat roost detection. For species emerging late at night during true darkness (e.g. *Plecotus* sp.; Rydell *et al.*, 1996), it is difficult to assess whether the bats acoustically detected actually emerge from the building surveyed, whereas species emerging early during dusk (*Pipistrellus* spp.) are easier to see leaving the building.

Our dataset did not permit us to directly test our prediction that maternity roosts of crevice‐dwelling species would be missed if only PRAs were carried out as too few maternity colonies were discovered during both PRAs and ERSs to permit taxon‐specific analysis. In our sample just under half of all maternity colonies, and 80% of all *Pipistrellus* spp. maternity colonies was missed during the PRA. This suggests that a taxon‐specific effect is likely, with *Pipistrellus* spp. maternity colonies at particular risk of being missed during a PRA. From a conservation perspective, this is a negative outcome as it implies that PRAs may sometimes fail to detect maternity colonies and ultimately lead to the destruction of the roost if ERSs are not prescribed and conducted. Even the destruction of one maternity colony could have a major impact on local bat populations, especially for rare species (Stone *et al*., 2013). We urge future studies to explore in greater detail the variation in detectability of maternity colonies among species guilds to provide adequate recommendations in terms of best‐practice sampling methods to adopt.

Our findings stress the importance of conducting both methods – PRA and ERSs – to detect the maximum number of species roosting in a building. When only a PRA is conducted, the inventory of bat species roosting in a building will be far from being exhaustive as crevice‐dwelling species are likely to be missed. The lack of ERSs to complement PRA may lead to erroneous conclusions and incorrect recommendations during the ecological impact assessment process. It is important to detect all bat roosts present during ecological impact assessment to comply with the legislation and enable development projects to run smoothly. Discovering bats at a later stage during a development project can cause unexpected delays, costs and design changes, which is detrimental for both bat conservation and developers.

### Detection probabilities during acoustic surveys

Regardless of the species, bat detection probabilities during acoustic surveys at a given site are known to vary depending on a range of factors, including weather conditions (Goerlitz, 2018), sampling duration (Skalak *et al*., 2012), number of observers/detectors deployed (Duchamp, Yates & Muzika, 2006) and sensitivity/directionality of detector types (Adams *et al.*, 2012). However, despite focusing on buildings with known bat occupancy to better identify sources of variations in bat detection probabilities in buildings using acoustic surveys, our findings revealed that none of the variables assessed significantly influenced bat detectability. This could be explained by the fact that reports we investigated seem to have strictly followed recommendations regarding weather conditions, number of surveyors, survey type and sampling duration given in the successive UK guidance for undertaking ERSs (Bat Conservation Trust, 2007; Hundt, 2012; Collins, 2016; see Appendix S1). Thus, our results would indirectly imply that these recommendations, albeit not evidence‐based, turn out to be appropriate to account for variations in bat detectability arising from sampling methodology. However, two points need to be acknowledged. First, one model assumption of the site‐occupancy models used (that occupancy state at each building is static over surveys within the sampling season) may not have been fulfilled and these results should be therefore be interpreted with caution. Second, we did not consider detector type in our models as we did not have information about the sensitivity of each device, which may vary considerably, even for the same brand depending on its use (Adams *et al.*, 2012). Consequently, another model assumption regarding homogeneity in bat detection may not have been entirely met.

### Recommendations

Where a bat roost survey is required to inform development control or European Protected Species licensing, we recommend that both a PRA and ERSs are required unless the PRA can eliminate the possibility of bat presence, that is, all areas can be accessed and searched thoroughly and no bats or evidence of bats is found. If there are cavities, cracks or crevices that cannot be searched then there is a possibility that, if only a PRA is carried out, bats or evidence of bats could be hidden, and species/roosts missed. This recommendation is in accordance with the current UK bat survey guidelines (Collins, 2016).

ERSs are primarily undertaken for two purposes, to confirm the absence of bats where a PRA is unable to eliminate the possibility of bat presence or, where a PRA has confirmed the presence of roosting bats, to gather further information about the number of bats present and their use of the structure. Here we provide recommendations for the former scenario. The number of ERSs specified by current UK guidelines depends on the suitability of the structure to be occupied by bats, as assessed by a consultant ecologist. While this study provides evidence of the number of ERSs required to confirm the absence of bats, it did not consider how the probability of detection is affected by the assessed suitability of a structure. However, our results suggest that for structures which require the greatest number of ERSs, that is, those that are assessed as highly suitable for roosting bats, and where a PRA has not definitively ruled out their presence, a minimum of at least three ERSs will be required to confirm the absence of *Pipistrellus *spp. and at least four ERSs to confirm the absence of *Plecotus* sp. The recommendation of three ERSs for *Pipistrellus* spp. is in accordance with current UK guidelines for highly suitable structures; however, the recommendation of four ERSs for *Plecotus* sp. exceeds the number of ERSs currently specified (Collins, 2016). In this study, the proportion of sites assessed using fewer than four ERSs was very high, representing 85% of the cases (Appendix S2). Nevertheless, where a thorough PRA can be carried out it is likely that *Plecotus* sp. would be discovered if present; in this study, *Plecotus* sp. were not discovered until the ERS stage (i.e. not discovered during the PRA) in only 14% of cases. A proportionate approach may see fewer visits carried out, for example if the results of these surveys would not impact on the subsequent action taken or for structures assessed to have lower suitability. Further work is required to investigate the influence of assessed suitability for bats on the optimum number of surveys to prove absence. Further work is also required for other species not included in this study. Finally, in line with Richardson *et al. *(2019), our results highlight the significant benefit of conducting evidence‐based research to test and improve survey methodology implemented in ecological impact assessment studies.

## Conflict of interest

The authors declare no conflicts of interest.

## Supporting information


**Appendix S1.** Histogram of (a) temperature data at sunset during emergence surveys and (b) sampling duration of emergence and re‐entry acoustic surveys conducted by ecological consultants. Data on the right side of the red dotted line are in accordance with recommendations made by the most recent UK guidance (Collins, 2016).

**Appendix S2.** Histogram showing the number of emergence and re‐entry surveys (ERSs) performed at the sites in our study. Sites on the right side of the blue dashed line meet our recommendation of three ERSs for *Pipistrellus* spp., and are in accordance with current UK guidelines for highly suitable sites. Sites on the right side of the red dashed line meet our suggested revised recommendation of four ERSs for *Plecotus* sp., which exceeds the number of ERSs currently specified with current UK guidelines (Collins, 2016).
