# Supplementary material for: Evaluating survey methods for bat roost detection in ecological impact assessment
Source: Anim Conserv. 2020 Apr 2;23(5):597–606. doi: 10.1111/acv.12574 (PMC7687239; doi:10.1111/acv.12574)
Supplement: Supplementary file 1 — Appendix S1. Histogram of (a) temperature data at sunset during emergence surveys and (b) sampling duration of emergence and re‐entry acoustic surveys conducted by ecological consultants. Data on the right side of the red dotted line are in accordance with recommendations made by the most recent UK guidance (Collins, 2016). [file ACV-23-597-s001.docx]

**Appendix S1.**


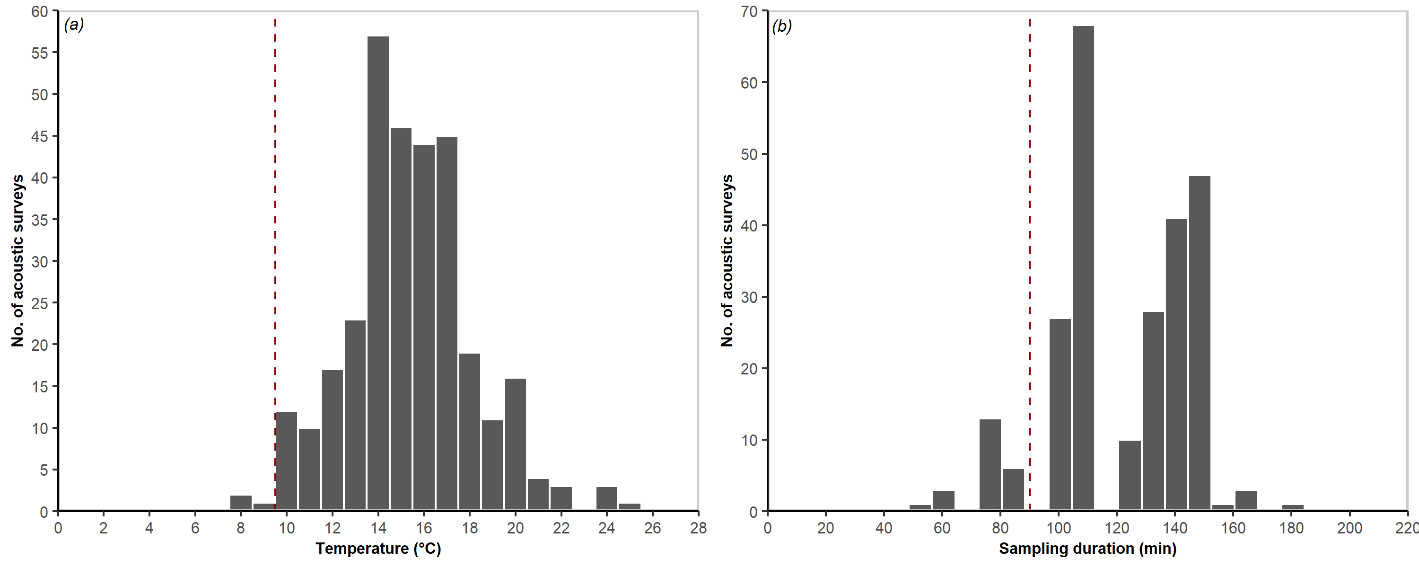


**Figure A1.** Histogram of (a) temperature data at sunset during emergence surveys and (b) sampling duration of emergence and re-entry acoustic surveys conducted by ecological consultants. Data on the right side of the red dotted line are in accordance with recommendations made by the most recent UK guidance (Collins, 2016).

**Reference**

Collins, J. (2016). Bat surveys for professional ecologists: good practice guidelines (3^rd^ edn). Bat Conservation Trust, London, UK.
