# Supplementary material for: Evaluating survey methods for bat roost detection in ecological impact assessment
Source: Anim Conserv. 2020 Apr 2;23(5):597–606. doi: 10.1111/acv.12574 (PMC7687239; doi:10.1111/acv.12574)
Supplement: Supplementary file 2 — Appendix S2. Histogram showing the number of emergence and re‐entry surveys (ERSs) performed at the sites in our study. Sites on the right side of the blue dashed line meet our recommendation of three ERSs for Pipistrellus spp., and are in accordance with current UK guidelines for highly suitable sites. Sites on the right side of the red dashed line meet our suggested revised recommendation of four ERSs for Plecotus sp., which exceeds the number of ERSs currently specified with current UK guidelines (Collins, 2016). [file ACV-23-597-s002.docx]

**Appendix S2.**


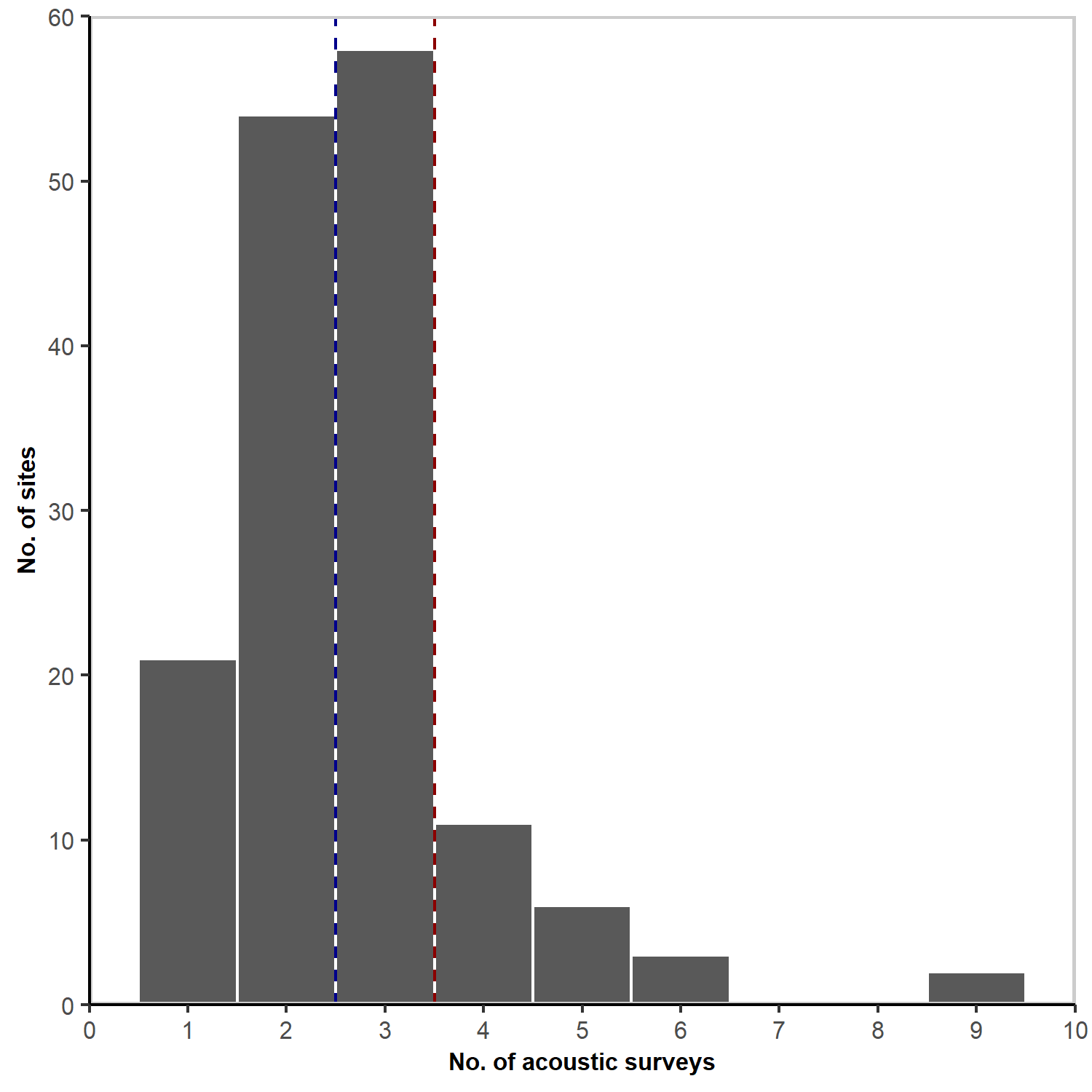


**Figure A2.** Histogram showing the number of emergence and re-entry surveys (ERSs) performed at the sites in our study. Sites on the right side of the blue dashed line meet our recommendation of three ERSs for *Pipistrellus* spp., and are in accordance with current UK guidelines for highly suitable sites. Sites on the right side of the red dashed line meet our suggested revised recommendation of four ERSs for *Plecotus* sp., which exceeds the number of ERSs currently specified with current UK guidelines (Collins, 2016).

**Reference**

Collins, J. (2016). Bat surveys for professional ecologists: good practice guidelines (3^rd^ edn). Bat Conservation Trust, London, UK.
